# Erosion of human X chromosome inactivation causes major remodeling of the iPSC proteome

**DOI:** 10.1016/j.celrep.2021.109032

**Published:** 2021-04-27

**Authors:** Alejandro J. Brenes, Harunori Yoshikawa, Dalila Bensaddek, Bogdan Mirauta, Daniel Seaton, Jens L. Hukelmann, Hao Jiang, Oliver Stegle, Angus I. Lamond

**Affiliations:** 1 Centre for Gene Regulation and Expression, School of Life Sciences, University of Dundee, Dow St., Dundee DD1 5EH, UK; 2 Cell Signalling & Immunology, School of Life Sciences, University of Dundee, Dow St., Dundee DD1 5EH, UK; 3 European Molecular Biology Laboratory, European Bioinformatics Institute, Wellcome Genome Campus, Hinxton CB10 1SD, UK; 4 Division of Cell Signalling, Fujii Memorial Institute of Medical Sciences, Institute of Advanced Medical Sciences, Tokushima University, 3-18-15 Kuramoto, Tokushima 770-8503, Japan; 5 Biosciences Core Labs, Proteomics, King Abdullah University of Science and Technology, Thuwal 23955-6900, Saudi Arabia; 6 Immatics Biotechnologies, Paul-Ehrlich-Str. 15, Tuebingen 72076, Germany; 7 European Molecular Biology Laboratory, Genome Biology Unit, Heidelberg, Germany; 8 Division of Computational Genomics and Systems Genetic, German Cancer Research Center, Heidelberg, Germany

**Keywords:** proteomics, iPSC, mass spectrometry, RNA-seq, X chromosome inactivation, gene expression, dosage compensation, erosion of X chromosome inactivation, proteome, transcriptome

## Abstract

X chromosome inactivation (XCI) is a dosage compensation mechanism in female mammals whereby transcription from one X chromosome is repressed. Analysis of human induced pluripotent stem cells (iPSCs) derived from female donors identified that low levels of XIST RNA correlated strongly with erosion of XCI. Proteomic analysis, RNA sequencing (RNA-seq), and polysome profiling showed that XCI erosion resulted in amplified RNA and protein expression from X-linked genes, providing a proteomic characterization of skewed dosage compensation. Increased protein expression was also detected from autosomal genes without an mRNA increase, thus altering the protein-RNA correlation between the X chromosome and autosomes. XCI-eroded lines display an ∼13% increase in total cell protein content, with increased ribosomal proteins, ribosome biogenesis and translation factors, and polysome levels. We conclude that XCI erosion in iPSCs causes a remodeling of the proteome, affecting the expression of a much wider range of proteins and disease-linked loci than previously realized.

## Highlights

• iPSCs with eroded XCI show defective dosage compensation at the protein level
• iPSCs with eroded XCI display elevated total protein content
• iPSCs with eroded XCI show increased ribosome and polysome levels
• Eroded XCI increases protein but not mRNA expression for 21% of autosomal genes

## Graphical abstract

**Figure:**
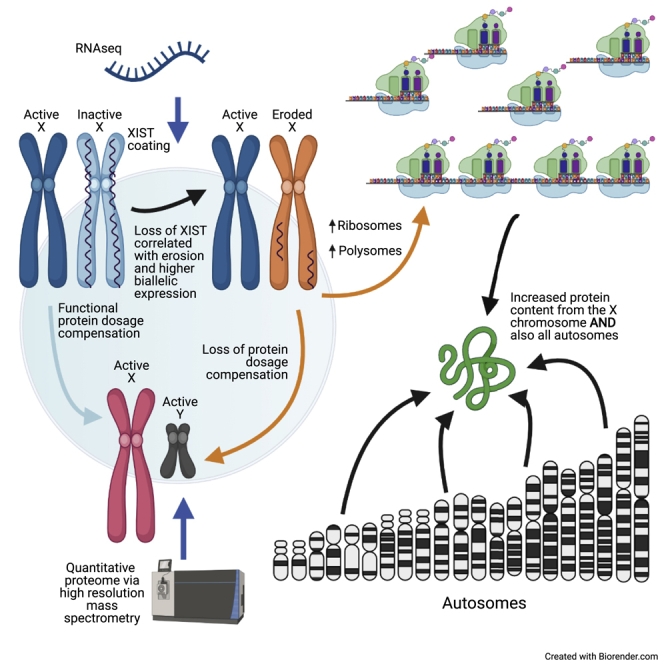

## Teaser

X chromosome inactivation (XCI) is a dosage compensation mechanism in female mammals. Brenes et al. show how erosion of XCI affects mRNA and protein expression in humans and uncover a major impact on global protein translation when XCI is eroded, affecting protein expression from autosomal as well as X-linked genes.

## Introduction

In humans and other mammalian species, female cells have two copies of the X chromosome, whereas males have a single X chromosome and a much smaller Y chromosome that is not present in females. In females, one of the two X chromosomes undergoes silencing, causing repression of transcription and thereby inactivating expression of alleles located on this second copy of the X. This process is termed X chromosome inactivation (XCI).

The XCI process in female cells is considered a critical dosage compensation mechanism that evolved in mammals as a way to equalize X-linked gene expression between males and females (Graves, 2016; Livernois et al., 2012). XCI is vital for embryonic development, and failure to induce XCI has been shown to cause embryonic lethality (Borensztein et al., 2017; Takagi and Abe, 1990). Furthermore, skewed XCI has also been shown to have major clinical consequences, with the emergence of numerous sex-specific genetic disorders, such as Rett’s syndrome (Lyst and Bird, 2015).

The initiation of XCI is controlled by a specific locus, termed the X-inactivation center (Xic) (Augui et al., 2011). The mechanism of XCI involves a profound structural reorganization of the inactivated copy of the X chromosome, which becomes heterochromatic and visibly compacted (Augui et al., 2011; Giorgetti et al., 2016). Within the Xic, a long, non-coding RNA, called “XIST,” has been shown to be an important component of the XCI process (Marahrens et al., 1997; Penny et al., 1996). Accumulation of XIST RNA across the inactive copy of the X chromosome triggers the changes that produce the transcriptionally inactive state (Dossin et al., 2020; Galupa and Heard, 2018).

Over a decade ago, breakthrough studies reported that terminally differentiated somatic cells could be reprogrammed back into a pluripotent state by the exogenous expression of a small set of transcription factors (Takahashi et al., 2007; Takahashi and Yamanaka, 2006; Yu et al., 2007). The resulting human induced pluripotent stem cells (iPSCs) were shown to share the hallmarks of their embryonic counterparts, including the induction of XCI (Wutz, 2012). However, for these cells, as well as for human embryonic stem cells (hESCs), XCI has been shown to be unstable in culture. Thus, some human primed iPSCs exhibit erosion of XCI, for which the X chromosome loses H3K27me3 marks, as well as XIST RNA expression (Anguera et al., 2012; Dandulakis et al., 2016; Mekhoubad et al., 2012). Although the role of XIST in relation to erosion of XCI remains unclear, it has been reported that the loss of XIST expression is characteristic of class III hESCs that display eroded XCI (Geens and Chuva De Sousa Lopes, 2017).

In this study, we explore the global consequences for human gene expression when XCI is eroded by using a collection of iPSCs derived from healthy female donors that were all reprogrammed from primary skin fibroblasts (Kilpinen et al., 2017). Specifically, we have analyzed the impact of XCI erosion by using 74 independent HipSci (https://www.hipsci.org) iPSC lines derived from female donors and also compared them to 46 lines derived from male donors by using both RNA sequencing (RNA-seq) and proteomic data (Mirauta et al., 2020). The data show that for our collection of iPSCs, a decrease in the expression of the lncRNA XIST was correlated with significantly higher biallelic expression, reflecting increased erosion of XCI.

We also report a global analysis comparing in parallel RNA and protein expression levels for lines that were stratified based upon having either high or low expression levels of XIST RNA. We provide an in-depth analysis of how erosion of XCI in human cells affects gene expression at the protein level. The data show that erosion of XCI increases both transcription and protein production from genes on the inactive X chromosome, and comparisons to the male lines show this erosion significantly affects dosage compensation at the protein level. Remarkably, we also uncover a widespread increase in the abundance of many proteins encoded by genes on the autosomes, independent of a parallel increase in transcription. Female cell lines with low levels of XIST RNA show a median increase of ∼13% in total protein content, along with higher levels of polysomes and components of the translational machinery. These data indicate that erosion of XCI can affect the expression of a much wider range of proteins and disease-linked gene loci than previously realized based on RNA analysis alone.

All of the raw and processed mass spectrometry (MS) files are available within PRIDE (Perez-Riverol et al., 2019; Vizcaíno et al., 2016) (PRIDE:PXD010557), and the RNA-seq data are available within ENA (Amid et al., 2020) (ENA:PRJEB7388).

## Results

### RNA-seq and proteomic datasets

**Figure 1:**
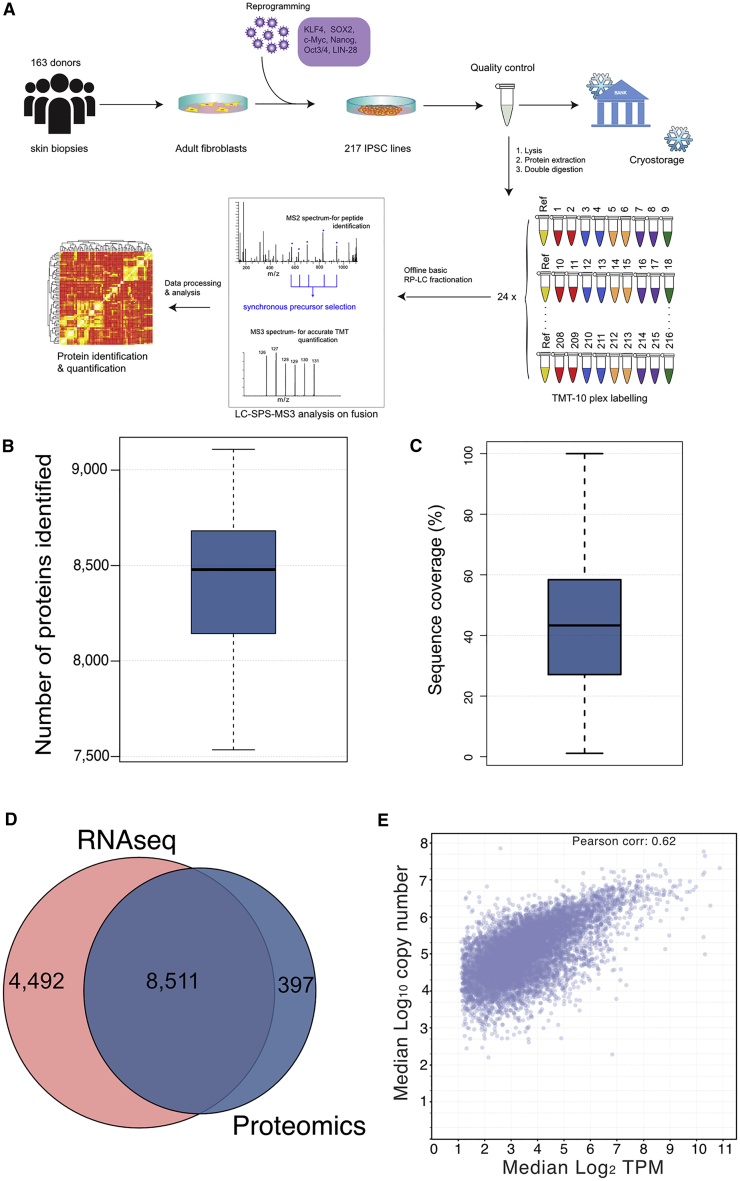
Comprehensive coverage For all boxplots, the top and bottom hinges represent the 1^st^ and 3^rd^ quartiles. The top whisker extends from the hinge to the largest value no further than 1.5 × interquartile range (IQR) from the hinge; the bottom whisker extends from the hinge to the smallest value at most 1.5 × IQR of the hinge. (A) The HipSci proteomics workflow from reprogramming to identification and quantification. (B) Boxplot showing the number of proteins identified per line across the 56 filtered (see STAR Methods) female iPSC lines. (C) Boxplot showing the sequence coverage for all proteins detected within the dataset. (D) Pie chart showing the overlap between quantified gene products in the proteomics and RNA-seq datasets. (E) Scatterplot comparing the median log_2_ transcripts per million (TPM) versus the median log_10_ copy number for all gene products.

All of the iPSC lines used for this study were generated by the HipSci project (https://www.hipsci.org). They were reprogrammed from human primary skin fibroblasts and subjected to rigorous quality-control procedures, which included array-based genotyping and gene expression profiling, as well as an evaluation of their pluripotency and differentiation properties (Kilpinen et al., 2017). This study analyzes gene expression data (Brenes et al., 2019; Mirauta et al., 2020) generated from 74 independent iPSC lines derived from healthy female donors and 46 lines derived from healthy male donors. The lines were grown using identical culture conditions, and aliquots were divided for parallel RNA-seq and proteomic analyses. The MS-based proteomic data were acquired using a tandem mass tag (TMT) workflow (see STAR Methods; Figure 1A).

The proteomic data for this study were processed using MaxQuant (Cox and Mann, 2008; Tyanova et al., 2016) and searched against the manually curated SwissProt database (The UniProt Consortium, 2017) with a 1% false discovery rate (FDR) threshold at the peptide spectrum match (PSM) and protein level (for more details see STAR Methods). Overall, it detected the expression of >9,500 protein groups (i.e., proteins/protein isoforms without discriminating peptides; hereafter termed proteins; Table S1), with a median of 8,479 proteins identified across all lines (Figure 1B), and a median protein sequence coverage of 42% across all proteins (Figure 1C). All downstream analyses were performed on a subset of 8,908 proteins, which were each identified with at least 3 “Razor + unique peptides” (RUP; ie., the number of unique peptides, plus the number of shared peptides used for the quantification of a protein; see STAR Methods). To compare protein expression levels between the respective iPSC lines, protein copy numbers were estimated using the “proteomic ruler” (Wiśniewski et al., 2014) approach and using the batch correction method previously described (Brenes et al., 2019; Plubell et al., 2017). This is well suited for the analysis of HipSci lines, which have been shown to have near-identical DNA content (Kilpinen et al., 2017).

From the RNA-seq data, after filtering, a total of 12,798 transcripts were quantified, (see STAR Methods; Table S1), with matching protein level data for 65% of them (Figure 1D). To explore the relationship between RNA and protein abundance levels in this set of iPSC lines, we calculated the Pearson correlation of mRNA abundance versus protein abundance (Figure 1E). This resulted in a Pearson correlation coefficient of 0.62, which is similar to that reported by multiple previous studies comparing mRNA and protein expression levels, both in different human cell types and for other mammalian species (Edfors et al., 2016; Lundberg et al., 2010; Ly et al., 2014).

### Erosion of XCI

**Figure 2:**
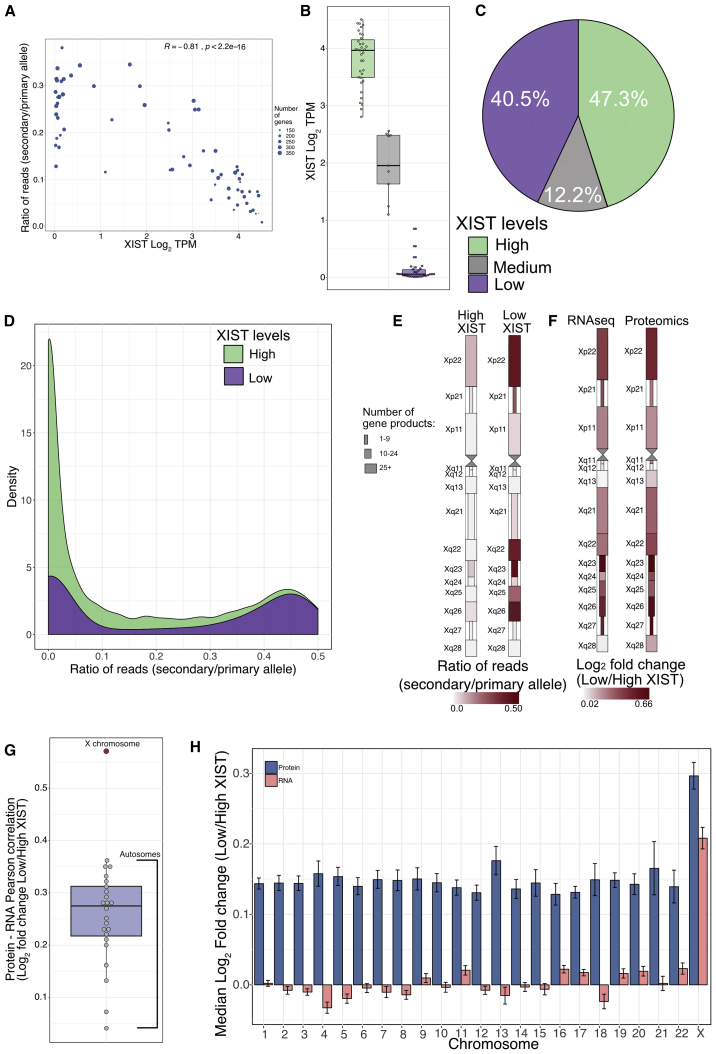
XIST and XCI For all boxplots, the bottom and top hinges represent the 1^st^ and 3^rd^ quartiles. The top whisker extends from the hinge to the largest value no further than 1.5 × IQR from the hinge; the bottom whisker extends from the hinge to the smallest value at most 1.5 × IQR of the hinge. (A) Scatterplot showing the ratio of reads derived from the secondary allele (lowest expressed allele) compared to the primary allele (highest expressed allele) for all X-linked transcripts versus the log_2_ XIST TPM for all healthy female lines. The size of the circle is determined by the number of transcripts used for the analysis. (B) Boxplot showing log_2_ TPM for the long non-coding RNA XIST across all 3 populations, namely, low, medium, and high XIST. (C) Pie chart showing the percentage of healthy female lines within each XIST-stratified population. (D) Stacked density plot for all X-linked gene products across all lines showing the ratio of reads mapped to the secondary allele compared to the primary allele for both the high and low XIST populations. (E) X chromosome map showing the ratio of reads derived from the secondary allele compared to the primary allele across chromosomal bands for both the high and low XIST populations. The size of the rectangles represents the number of gene products per band. (F) X chromosome map showing the log_2_ fold change (low/high XIST) across chromosomal bands for both the RNA-seq and proteomic datasets. The size of the rectangles represents the number of gene products per band. (G) Boxplot showing the Pearson correlation coefficient comparing log_2_ fold change (low/high XIST) at the RNa-seq and proteomics level for all chromosomes. Autosomes are colored in gray; the X chromosome is colored in red. (H) Bar plot showing the median log_2_ fold change (low/high XIST) for all gene products aggregated at the chromosome level for both RNA-seq and proteomics. The error bars represent the SEM.

As it had been previously reported that there was a correlation between the loss of XIST RNA coating the inactive X chromosome and erosion of XCI, we first evaluated the relationship between the levels of XIST RNA expression and the erosion of XCI within the HipSci iPSC lines, as measured by an allele-specific expression (ASE) analysis on X-linked genes (Figure 2A). This showed a clear correlation between iPSC lines with low XIST expression and increased levels of biallelic expression for X-linked genes. Interestingly, a parallel analysis focused on XACT RNA, another long non-coding RNA implicated in the mechanism of XCI in humans (Vallot et al., 2017), showed little to no correlation with altered biallelic expression in these iPSC lines (Figure S1).

Next, the 74 iPSC lines were stratified into low, medium, and high XIST RNA populations, based on the RNA-seq expression data (Figure 2B; Table S2). This identified two main populations, with 47.3% showing the expected high levels of XIST RNA and a surprisingly elevated proportion, 40.5%, of the iPSC lines having very low levels of XIST RNA expression (Figure 2C). A minor population (12.2%) that showed an intermediate level of XIST expression was also identified. However, as this population represented a low number of iPSC lines it was not used for the main downstream analysis. The rest of the study focused on comparing gene expression specifically between the populations stratified by high and low levels of XIST RNA expression.

We next set out to examine the relationship between XIST expression levels and allelic expression within X-linked gene products (Figure 2D). This analysis showed that iPSC lines with high levels of XIST expression had a significantly lower (p = 2.2e^−16^) fraction of genes, with reads derived from the lowest expressed allele (hereafter termed secondary allele; see STAR Methods), compared to the low XIST population. Gene products within iPSC lines displaying high levels of XIST had a median of 99.5% of reads originating from the highest expressed allele (hereafter termed primary allele; see STAR Methods), with only 0.5% from the secondary allele. iPSC lines with low levels of XIST showed an increase in the proportion of reads derived from the secondary allele, with a median of 22.6%, and with 77.4% of reads derived from the primary allele. We therefore conclude that XIST expression levels provide a suitable marker for detecting erosion of XCI within the iPSC lines analyzed.

We next mapped all the X-linked genes to their respective bands within the X chromosome and studied allele expression for genes across all bands, for both the high and low XIST populations (Figure 2E). These data again emphasize that the population with high XIST expression has much lower biallelic expression than the low XIST population. However, it was apparent that the level of biallelic expression is not uniform across the X chromosome. Even within the high XIST population, certain bands, such as Xp22, Xq23, and Xq26, are more prone to displaying increased expression from the secondary allele (Figure 2E). These same bands also displayed higher biallelic expression within the low XIST population.

We expanded the chromosomal band analysis by calculating the median log_2_ fold change between the high and low XIST populations, for all gene products and across all bands, at both the RNA-seq and proteomics levels (Figure 2F). As seen for the allelic expression, the fold change across chromosomal bands was not uniform. Interestingly, some of the hotspots highlighted by the allelic analysis (e.g., bands Xp22, Xq23, and Xq26) were also among the sites showing highest levels of change in RNA and protein expression. This is consistent with previous observations showing that there are specific loci that can preferentially escape XCI (Balaton and Brown, 2016; Tukiainen et al., 2017). We note that the independent transcriptomic and proteomic datasets both displayed very similar patterns of gene expression variation in response to XIST levels across the X chromosome. This concordance in RNA- and protein-level data is consistent with a predominantly transcription-driven regulation of X-linked gene expression.

We also wanted to understand how the changes in gene expression between the high and low XIST populations behaved for genes across all other chromosomes. Hence, we used all gene products that were detected both in both the RNA-seq and proteomics datasets, aggregated them at the chromosome level, and compared their respective RNA and protein fold changes. This chromosome-specific view showed that the highest fold change concordance is observed within X-linked genes, with a Pearson correlation of 0.56 (Figure 2G). However, this same level of concordance was not observed across all other chromosomes, as each of the autosomes had a much lower correlation coefficient than was seen for the X chromosome, with the second highest being chromosome 10 with a correlation coefficient of 0.36 and the median being 0.27. The data thus indicate a difference between X-linked genes and genes on all of the autosomes.

To quantify these differences, we compared the median fold change for RNAs and proteins across all chromosomes (Figure 2H). Unsurprisingly, the highest median increase observed within the low XIST, compared to the high XIST population, at both the RNA and protein levels, occurs for genes on the X chromosome. However, the proteomics data, unlike the RNA-seq data, also displayed increased median fold changes across all other chromosomes as well.

In summary, the RNA expression data show that iPSC lines with high levels of XIST RNA display significantly lower biallelic expression than the iPSC lines with low levels of XIST, with 99.5% of the reads derived from the primary allele and only 0.5% from the secondary allele. The lines with low expression of XIST showed a higher proportion of reads derived from the secondary allele (22.6%), consistent with erosion of XCI. The data also show that erosion of XCI in iPSCs results in both increased transcription and protein expression for X-linked genes. However, the same was not observed for genes on the autosomes, for which the increased median fold change seen across all chromosomes was detected only at the protein level.

### Impact of XCI erosion on the autosomes and dosage compensation

**Figure 3:**
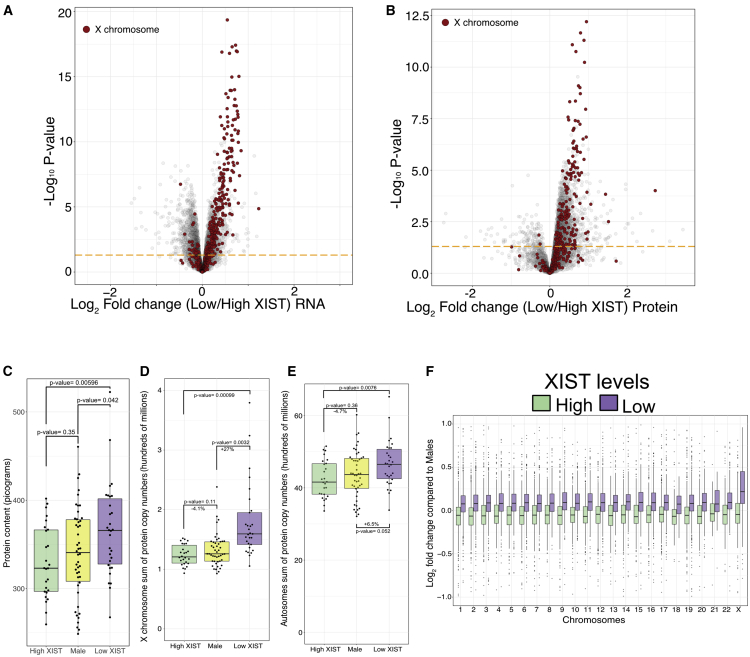
Multi-omic overview For all boxplots, the bottom and top hinges represent the 1^st^ and 3^rd^ quartiles. The top whisker extends from the hinge to the largest value no further than 1.5 × IQR from the hinge; the bottom whisker extends from the hinge to the smallest value at most 1.5 × IQR of the hinge. (A) Volcano plot showing the log_2_ fold change (low/high XIST) on the x axis, with the −log_10_ p value on the y axis for the RNA-seq dataset. X chromosome transcripts are highlighted in red; autosome transcripts are colored gray. All transcripts above the orange line have a p value lower than 0.05. (B) Volcano plot showing the log_2_ fold change (low/high XIST) on the x axis, with the −log_10_ p value on the y axis for the proteomic dataset. X chromosome proteins are highlighted in red; autosomal proteins are colored gray. All proteins above the orange line have a p value lower than 0.05. (C) Boxplot showing the estimated protein content (see STAR Methods) for the high XIST, low XIST, and male populations. (D) Boxplot showing the sum of protein copy numbers across the X chromosome for the high XIST, low XIST, and male lines. (E) Boxplot showing the sum of protein copy numbers across all autosomes for the high XIST, low XIST, and male lines. (F) Boxplot showing the median protein log_2_ fold change (high XIST/Males and low XIST/Males) across all chromosomes.

Next, we focused on a differential expression analysis, comparing the high versus low XIST-stratified populations at both the RNA and protein levels. This analysis showed that ∼55% of X-linked genes in the low XIST population exhibited significantly increased expression of both RNA and protein, as compared to the high XIST population (Figures 3A and 3B; Table S3). However, when comparing gene expression from autosomes, once again, we detected differences between the RNA and protein datasets. Thus, 9% of autosomal transcripts (1,087 out of 12,042) were significantly increased in expression and 11.2% (1,344 out of 12,042; Table S4) were significantly decreased in expression in the low XIST compared to the high XIST population. In contrast, the proteomics data showed that 27.8% of the quantified autosome-encoded proteins (2,383 out of 8,593; Table S4) were significantly increased in expression in the low XIST population, whereas only 1.2% of autosome-encoded proteins (107 out of 8,593) were significantly decreased in expression (Figure 3B). These results show that there is a clear effect within the proteomics data that is not recapitulated by the RNA-seq data.

Based on the increased fold change across all chromosomes and significantly increased expression of over 2,300 autosomal proteins, we suspected that the low XIST population of iPSC lines may have a higher average protein content per cell, as compared to the high XIST population. To test this hypothesis, we used the MS data to estimate the total protein content and compare both XIST-stratified populations to each other. Furthermore, this dataset also provided an opportunity to study the impact of human dosage compensation at the protein level and thus to determine how the global proteome may respond to erosion of XCI. Hence, we also compared both XIST-stratified populations to 46 iPSC lines derived from healthy male donors (see STAR Methods).

No significant differences in total protein content were detected between the high XIST female lines and the male lines (Figure 3C). However, the low XIST population had a significant increase (p = 0.042) of 7.3% in total protein content compared to the male lines and an even more pronounced increase (p = 0.006) of 13.2% compared to the high XIST lines (Figure 3C). To check if these changes in protein content were related to potential cell cycle differences between the respective stratified populations, we analyzed the expression of a panel of genes previously characterized as being cell cycle regulated (Ly et al., 2014). Gene expression at both the RNA and protein levels showed no significant differences for these known cell-cycle-regulated genes between the high and low XIST populations (Figure S2). Hence, we conclude that the observed differences in protein content linked with erosion of XCI are likely not the result of altered kinetics of cell cycle progression.

We drilled down on these comparisons further and focused on the total copy numbers for all X-linked proteins across both XIST-stratified female populations and the male lines. Once again, this comparison revealed no significant differences between the high XIST female population and the males (Figure 3D). In contrast, the low XIST population saw a dramatic increase in protein copy numbers of 27% (p = 0.0032) compared to the males (Figure 3D). When we repeated this analysis for autosomal proteins (Figure 3E), the low XIST female population displayed 6.5% higher (p = 0.052) protein copy numbers than the males and 11.8% higher (p = 0.00099) protein copy numbers than the high XIST female population. Hence, these data suggested there was little or no significant difference in total protein levels between the high XIST female population and iPSC lines from male donors, whereas the low XIST female population was significantly different from both. The medium XIST population appeared to be more closely aligned to the high XIST than to the low XIST population (Figure S3)

To obtain a more granular view, we compared the changes in expression for each protein within both the high and low XIST female populations, in comparison to the male population, for each of their respective chromosomes (Figure 3F). When comparing the male to high XIST female lines, we found no significant fold change difference between the X chromosome and any other chromosome. This finding demonstrates that XCI is effective at ensuring similar expression levels of X linked proteins between males and females with robust XCI. However, the situation is different when comparing male-derived lines with low XIST female lines, for which the median fold change of proteins encoded by genes on the X chromosome is significantly higher than for genes on all other chromosomes.

### Proteome-specific response to XCI

**Figure 4:**
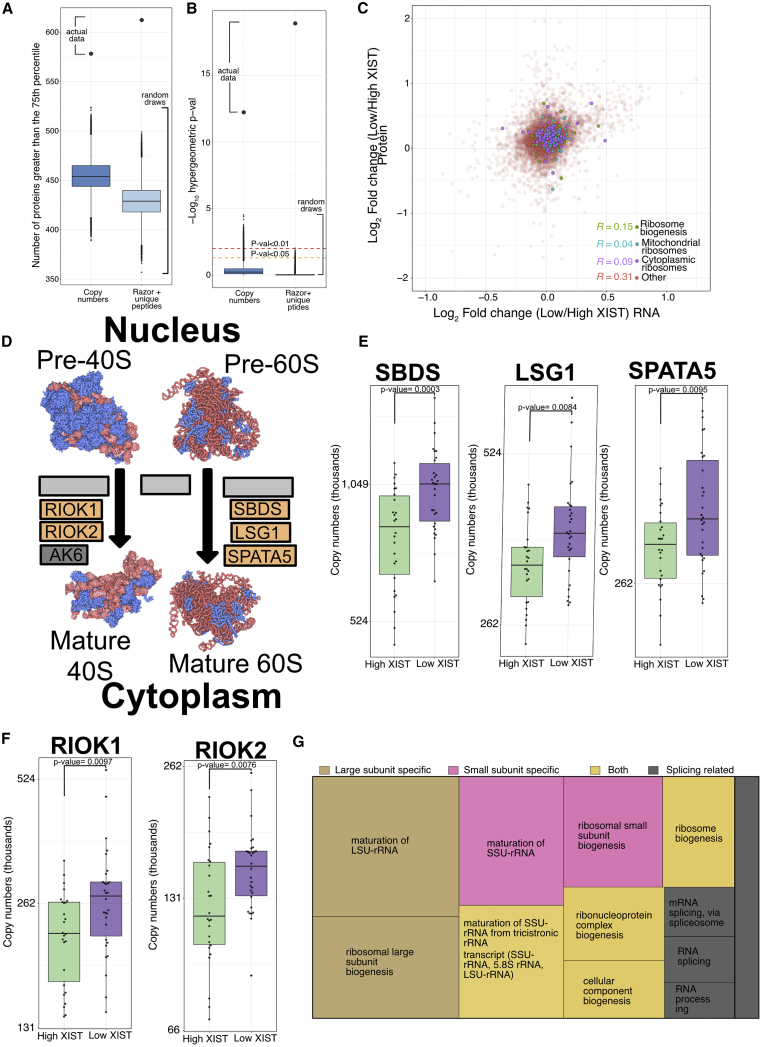
Ribosome biogenesis For all boxplots, the bottom and top hinges represent the 1^st^ and 3^rd^ quartiles. The top whisker extends from the hinge to the largest value no further than 1.5 × IQR from the hinge; the bottom whisker extends from the hinge to the smallest value at most 1.5 × IQR of the hinge. (A) Boxplot showing the number of proteins with copy numbers greater than the 75^th^ percentile and Razor + unique peptides greater than the 75^th^ percentile for the simulations and the actual experimental data (see STAR Methods). (B) Boxplot showing the hypergeometric p value for proteins with copy numbers greater than the 75^th^ percentile and Razor + unique peptides greater than the 75^th^ percentile for the simulations and the actual experimental data (see STAR Methods). (C) Scatterplot showing the log_2_ fold change (low/high XIST) at the protein and RNA level. Ribosome biogenesis and cytoplasmic and mitochondrial ribosomal proteins are highlighted, and Pearson correlation coefficients are provided. (D) Schematic showing the cytoplasmic ribosome biogenesis proteins with proteins significantly increased in expression highlighted in orange. (E) Boxplot showing the protein copy numbers for SBDS, LSG1, and SPATA5 within the low and high XIST populations. (F) Boxplot showing the protein copy numbers for RIOK1 and RIOK2 within the low and high XIST populations. (G) Treemap plot showing the results of a biological process overrepresentation test focused on ribosome biogenesis proteins. The rectangle size is proportional to the enrichment level of the specific terms.

We next investigated in more detail how the proteome was altered in the low XIST population and examined which types of proteins and which protein functions showed changes. We focused on proteins that showed significantly increased expression (q < 0.05) in the Low XIST population compared to the high XIST population, but without a corresponding significant increase in the RNA-seq data. We discovered that this group of proteins were robust identifications, as they were enriched in high abundance proteins (abundance greater than the 75^th^ percentile; see STAR Methods), with high numbers of RUPs detected (RUP greater than the 75^th^ percentile; Figures 4A and 4B).

A follow-up analysis, focused on biological processes, was carried out by a Gene Ontology (GO) overrepresentation test, using Panther (Mi et al., 2017). This analysis showed that these proteins were enriched specifically for the GO terms “ribonucleoprotein complex biogenesis” and “mRNA metabolic process.” We note that these enriched GO terms are associated with many proteins involved in post-transcriptional mechanisms that could increase total protein expression from a constant amount of mRNA. This includes genes related to processes including ribosome subunit biogenesis, ribosome function, and the control of protein translation. Consistent with this result, when comparing changes in expression at the respective RNA and protein levels between the XIST stratified populations, we found that Pearson correlation coefficients were particularly low for the ribosomal (0.09) and ribosome subunit biogenesis (0.15) proteins (Figure 4C). This finding indicates a potentially important role for post-transcriptional mechanisms in regulating protein expression from these genes.

Overall, >36% of all proteins involved in ribosome subunit biogenesis, as described in KEGG (Kanehisa and Goto, 2000), showed significantly increased (q < 0.05) expression within the low XIST population, and of these proteins, >70% of those involved in the cytoplasmic stages of ribosome subunit biogenesis showed increased protein expression (Figure 4D). For example, SPATA5 (a human homolog of yeast Drg1; p = 0.0095), SBDS (p = 0.0003), and LSG1 (p = 0.0084) (Figure 4E), which are all involved in the final step of 60S maturation and the atypical RIO kinases RIOK1 (p = 0.0097) and RIOK2 (p = 0.0076; Figure 4F), involved in the final step of 40S maturation (Vanrobays et al., 2003), all showed significantly increased protein expression within the low XIST population. Interestingly, all of these proteins are involved in the cytoplasmic quality control of ribosomes (Cerezo et al., 2019; Karbstein, 2013; Peña et al., 2017). To drill down further into the process of ribosome subunit biogenesis, we performed a more granular enrichment analysis on the proteins involved within this pathway and found the highest enrichment was on terms related to large subunit biogenesis and ribosomal RNA (Figure 4G)

### Proteome-specific changes affecting ribosomes and translation initiation

**Figure 5:**
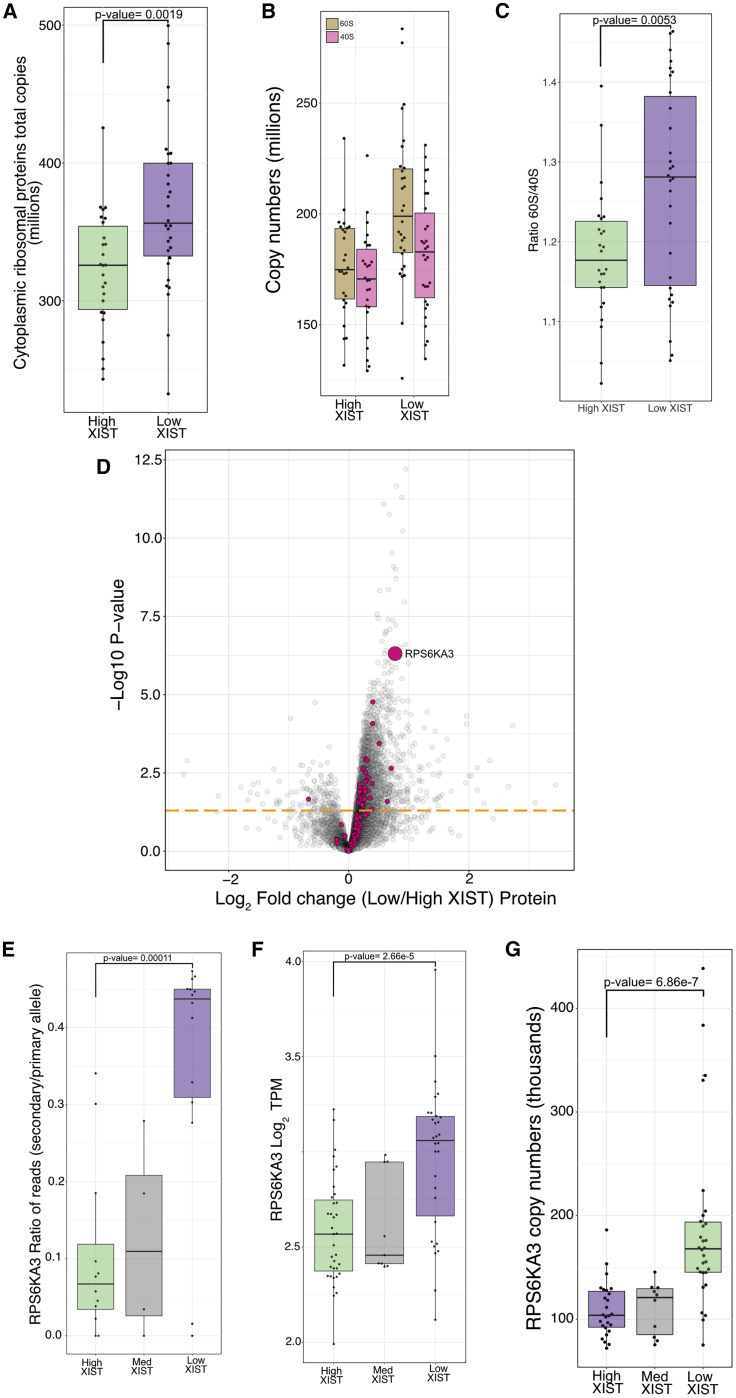
Ribosomes and translational initiation For all boxplots, the bottom and top hinges represent the 1^st^ and 3^rd^ quartiles. The top whisker extends from the hinge to the largest value no further than 1.5 × IQR from the hinge; the bottom whisker extends from the hinge to the smallest value at most 1.5 × IQR of the hinge. (A) Boxplot showing the copy numbers for the sum of all cytoplasmic ribosomal proteins within the high and low XIST populations. (B) Boxplot showing the copy numbers for the sum of all 60S (large ribosomal subunit) and 40S (small ribosomal subunit) proteins within the high and low XIST populations. (C) Boxplot showing the ratio of the sum of 60S to 40S ribosomal proteins within the high and low XIST populations. (D) Volcano plot showing the protein log_2_ fold change (low/high XIST) on the x axis, with the −log_10_ p value on the y axis. Ribosomal proteins and ribosomal S6 kinases are highlighted in pink; all other proteins are colored gray. All proteins above the orange line have a p value lower than 0.05. (E) Boxplot showing the ratio of reads mapped to the secondary allele compared to the primary allele for RPS6KA3 within the High, Medium and Low XIST populations. (F) Boxplot showing the log_2_ TPM of RPS6KA3 within the high, medium, and low XIST populations. (G) Boxplot showing the protein copy numbers of RPS6KA3 within the high, medium, and low XIST populations.

When we focused on the total estimated copy numbers for the cytoplasmic ribosomes, we noticed they mirrored the protein content closely, with a mean increase of 13% (p = 0.0019) in the low XIST population (Figure 5A). Interestingly, the changes were not uniform between the large subunit (60S) and the small subunit (40S). The largest increase in the low XIST population affected proteins belonging to the 60S (Figure 5B), resulting in a significant change (p = 0.0053) in the ratio of protein copy numbers between 60S and 40S ribosomal proteins (Figure 5C). Overall, ∼42% of the ribosomal proteins and ribosomal S6 kinases detected were significantly increased in expression in the low XIST population (Figure 5D). Of these proteins, the one with the highest p value (p < 6.86e^−07^) was p90 ribosomal S6 kinase (RPS6KA3), which is encoded on the X chromosome and is linked to cell growth by increased cap-dependent translation through phosphorylation of RPS6 (Roux et al., 2007) and RPTOR (Carrière et al., 2008; for all significantly increased X-linked kinases see Figure S4).

As RPS6KA3 is an X-linked kinase, we checked the ratio of reads mapped to the secondary allele compared to the primary allele and detected significant differences between the high and low XIST lines (p = 0.00011). Thus, the median ratio for the high XIST population was 0.07, whereas the median ratio in the low XIST population was 0.44 (Figure 5E). We also detected a significantly higher expression of RPS6KA3 within the low XIST compared to the high XIST population, in both the RNA-seq (Figure 5F) and proteomics (Figure 5G) datasets. These data support a model in which transcriptional derepression of the inactive X chromosome in low XIST lines increases the expression of proteins encoded on the X, which in turn can increase mRNA translation and protein expression for autosomal genes.

**Figure 6:**
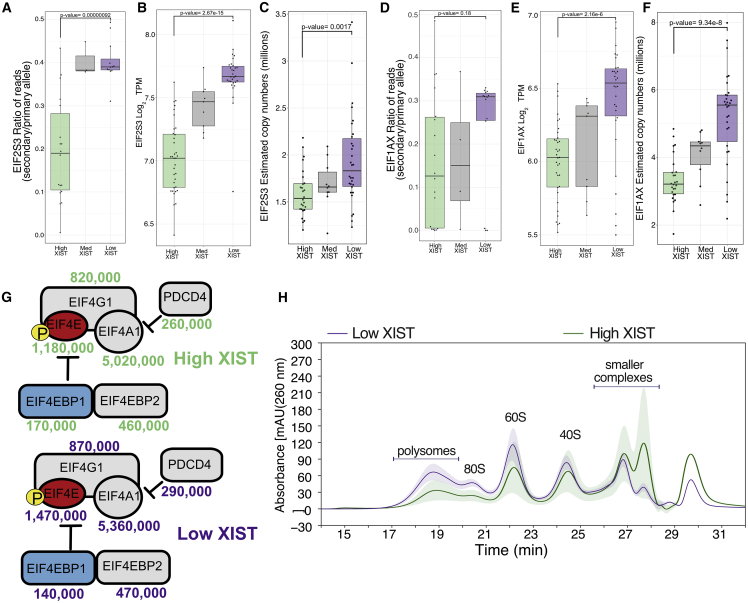
Translational machinery For all boxplots, the bottom and top hinges represent the 1^st^ and 3^rd^ quartiles. The top whisker extends from the hinge to the largest value no further than 1.5 × IQR from the hinge; the bottom whisker extends from the hinge to the smallest value at most 1.5 × IQR of the hinge. (A) Boxplot showing the ratio of reads mapped to the secondary allele (lowest expressed allele) compared to the primary allele (highest expressed allele) of EIF2S3 within the high, medium, and low XIST populations. (B) Boxplot showing the log_2_ TPM for EIF2S3 within the high, medium, and low XIST populations. (C) Boxplot showing the protein copy numbers for EIF2S3 within the high, medium, and low XIST populations. (D) Boxplot showing the ratio of reads mapped to the secondary allele compared to the primary allele for EIF1AX within the high, medium, and low XIST populations. (E) Boxplot showing the log_2_ TPM of EIF1AX within the high, medium, and low XIST populations. The bottom and top hinges represent the 1^st^ and 3^rd^ quartiles. (F) Boxplot showing the protein copy numbers of EIF1AX within the high, medium, and low XIST populations. (G) Schematic showing the protein copy numbers for the eIF4F complex and its inhibitors displayed for both the high and low XIST populations. Proteins represented by red boxes are significantly increased, light blue boxes are significantly decreased, and elements in gray boxes remain unchanged. (H) Ribo-Mega-SEC-derived line plot showing the mean high and low XIST polysome profile, with the colored ribbon representing the standard deviation.

We next looked for additional X-linked gene products with the potential to affect mRNA translation, focusing on the translation initiation factors EIF2S3 and EIF1AX. These proteins both form part of the 43S translation preinitiation complex (Jackson et al., 2010). EIF2S3 is a member of the heterotrimeric eIF2 complex, which delivers an initiator methionyl transfer RNA to the ribosome. Based on the protein expression data in this study, EIF2S3 appears to be the rate-limiting subunit of the eIF2 complex within the iPSC lines (Figure S5). As seen with RPS6KA3, a significantly higher ratio of reads from the secondary allele of EIF2S3 are seen within the low XIST compared to the high XIST population (p = 9.2e^−7^; Figure 6A), together with significantly higher expression at the RNA-seq (p = 2.67e^−15^; Figure 6B) and proteomics (p = 0.0017; Figure 6C) levels. Similarly, EIF1AX is involved in virtually all the steps in mRNA translation initiation, from the pre-initiation to ribosomal subunit joining (Nag et al., 2016), and it shows a dramatic median increase of over 2.3 million protein copies in the low XIST population. A higher median fraction of reads was seen from the EIF1AX secondary allele (0.31 in the low XIST versus 0.13 in the high XIST; Figure 6D). However, this difference did not meet a statistically significant threshold, likely due to outlier lines within the high XIST population with a high ratio of reads from the secondary allele. Nonetheless, the expression levels of EIF1AX at both the RNA-seq (p = 2.16e^−6^; Figure 6E) and proteomics (p = 9.34e^−8^; Figure 6F) levels were significantly increased in the low XIST population. To validate further the MS-based identifications and quantifications, we mapped the peptide coverage for all 3 X-linked translational modulators (i.e., RPS6KA3, EIF2S3, and EIF1AX), which revealed robust results with high numbers of RUPs (all over 10 RUP) detected, as well as high sequence coverage (all over 53%; Figure S6).

The potential impact of eroded XCI on factors affecting the efficiency of translation initiation was not limited to an upregulation of proteins encoded by genes on the X chromosome only. For example, eIF4F is another complex that is vital for translation initiation (Merrick, 2015). It is composed of the alpha (EIF4A1 and EIF4A3), epsilon (EIF4E), and gamma (EIF4G1 and EIF4G3) subunits. The stoichiometry of EIF4E, the cap-binding subunit (located on chromosome 4), to its inhibitors EIF4EBP1 (located on chromosome 8) and EIF4EBP2 (located on chromosome 10) has been proposed as one of the regulatory mechanisms for eIF4F translational control (Richter and Sonenberg, 2005). Interestingly, in the low XIST population, there is a significant increase (p = 0.0006) in EIF4E levels compared to the high XIST population, alongside a parallel significant decrease (p = 0.007) in the levels of EIF4EBP1 (Figure 6G). The decrease in EIF4EBP1 is particularly relevant in this case, as only ∼1.2% of all proteins are significantly decreased in expression in the low XIST population.

In summary, the data are consistent with erosion of XCI in the low XIST population, causing a major change in the proteome that results in a global increase in total protein levels, which is mediated, at least in part, by increased levels of translation. To test the hypothesis that the low XIST population has increased translational capacity, we compared polysome and ribosome profiles between iPSC lines showing either high or low levels of XIST RNA by using the Ribo Mega-size exclusion chromatography (SEC) method (Yoshikawa et al., 2018). This SEC-based method can separate large protein complexes, including polysomes, 80S monosomes, and ribosome subunits.

The Ribo Mega-SEC data were remarkably consistent with the MS-based proteomics data. This result showed that both the polysome-containing fractions and 80S-monosome-containing fractions are increased significantly in extracts from iPSC lines with low levels of XIST RNA compared to extracts from iPSC lines with high levels of XIST RNA (Figure 6H). Moreover, the SEC data show a more pronounced increase within the low XIST population in the ratio of the 60S to the 40S ribosome subunits, consistent with a larger increase in 60S ribosomal protein levels in the MS data (Figures 5B and 5C). The polysome fractionation data are thus consistent with a model in which erosion of XCI in the iPSC lines expressing low levels of XIST RNA upregulates the protein translation capacity and leads to a global increase in protein levels from multiple genes on both autosomes and the X chromosome.

## Discussion

This study provides an in-depth global analysis of how erosion of X chromosome inactivation (XCI) affects gene expression and dosage compensation at the protein level in human cells and compares this analysis to matching RNA-seq data. We analyzed RNA expression in 74 independent human iPSC lines derived from healthy female donors that were generated by the HipSci consortium (Kilpinen et al., 2017). This analysis showed that ∼40% of these lines expressed very low levels of XIST, a long non-coding RNA vital for the establishment of XCI (Marahrens et al., 1997; Penny et al., 1996). Further analysis of allelic expression from X-chromosome-encoded genes showed that low levels of XIST RNA strongly correlated with erosion of XCI, as reflected by a significantly increased fraction of reads being mapped to the lowest expressed (secondary) allele (higher biallelic expression). We therefore characterized how the erosion of XCI remodels gene expression and the human proteome by comparing in detail the levels of RNA and protein expression across 56 female iPSC lines, stratified according to high versus low XIST RNA levels, as well as comparing them to 46 lines derived from healthy male donors, which have only 1 X chromosome.

First, the data show that the low XIST population significantly upregulated the expression of many gene products on the X chromosome at both RNA and protein levels. Considering the evidence of increased biallelic expression within that same population, these data indicate that erosion of XCI causes increased protein expression from X-linked genes, primarily by a transcriptional mechanism.

We also leveraged the distinct characteristics of our dataset to compare how protein expression levels are affected by dosage compensation in the respective high and low XIST-stratified female lines versus the male lines. The data indicate that compared to the males, the high XIST lines, which exhibit robust XCI, show similar patterns of expression for genes derived from the X chromosome. This result suggests that when XIST levels are high, there is effective dosage compensation for the extra X chromosome copy, acting at the protein level. However, for the lines with low XIST expression levels, which exhibit higher erosion of XCI, a very different situation is evident. In this case, the total copies of X-chromosome-encoded proteins are increased in expression by 27% compared to males, which makes the gene expression from the X chromosome significantly different between males and females.

Second, we also detected a significant increase in protein expression levels from 26% of autosomal genes within the low XIST female lines. In contrast to genes on the X chromosome, 21% of all autosomal gene products were increased only at the protein level and not the RNA level. Thus, autosome-encoded genes showed a low overall RNA:protein fold change correlation (median Pearson correlation for autosomes of 0.27, compared with 0.56 for the X chromosome).

It should be noted that, unlike the RNA-seq data, protein copy numbers can be estimated from the MS data by the proteomic ruler without the need for spike-ins (Wiśniewski et al., 2014). The estimated copy numbers allow us to calculate and explore differences in absolute protein content. With this RNA-seq dataset and normalization approach, which did not include spike-in controls, it is not possible to detect potential transcriptional amplification effects (Lovén et al., 2012). Furthermore, recent reports have suggested that in mESCs, changes in ERK signaling can cause hypomethylation (Choi et al., 2017; Song et al., 2019), potentially also affecting global transcription. Our data reveal that multiple X-linked kinases associated with ERK signaling are significantly increased in expression within the low XIST population (Figure S4). Their contribution to cell phenotypes here remains to be determined. Therefore, we cannot completely exclude the possibility that absolute changes at the transcript level may also occur for autosomal genes, which would be masked due to technical issues in the detection of transcriptional amplification. Moreover, although erosion of XCI is generally thought to specifically affect the expression of X-linked genes, we note that increased transcription from autosomal genes has been reported in murine trophoblasts when they failed to induce XCI (Sakata et al., 2017). Nonetheless, the data from our study indicate that erosion of XCI in human cells can affect protein levels encoded by a much wider range of genes than was previously shown by RNA-seq data alone, including autosome-linked genes and disease loci.

Third, the comparison of high versus low XIST iPSC populations showed that the low-XIST-expressing cell lines had a median increase of ∼13% in total protein content, which was also significantly higher than the protein content of the lines derived from healthy male donors. Considering potential mechanisms that could cause this increased protein content, we found several lines of evidence that suggest it may result, at least in part, from post-transcriptional regulation affecting the translation efficiency of a subset of mRNAs. It has been proposed that translation rates positively correlate with protein abundance (Brockmann et al., 2007; Liu et al., 2016; Marguerat et al., 2012). Congruently, our data show the autosome-encoded proteins that are significantly increased in expression, but without a corresponding mRNA increase, are enriched in high abundance proteins with high peptide counts.

Focusing on the proteins that show statistically significant, RNA-independent increases in expression in low XIST population revealed an enrichment for GO terms associated with ribonucleoprotein complex biogenesis. Furthermore, by conducting independent polysome profiling analyses comparing high and low XIST iPSC lines, using the Ribo Mega-SEC method (Yoshikawa et al., 2018), we also showed a significant increase in the polysomes and 80S ribosomes within the low XIST lines. Thus, analyses using both the separate MS proteomics and polysome methods, support the view that iPSCs derived from healthy female donors showing XCI erosion have increased protein translation activity, resulting in a global increase in total protein levels. It will be interesting to analyze in the future whether mRNAs encoding the subset of autosomal proteins that show increased abundance share some common features or sequence motifs that promote efficient translation.

In light of the elevated protein content and polysome levels observed in the low XIST iPSC population, it is interesting that two X-linked genes that encode important regulators of translational initiation (EIF1AX and EIF2S3), as well as a kinase known to modulate translation (RPS6KA3), all show highly increased expression at both the RNA and protein levels. It has been proposed that protein synthesis is principally regulated at the initiation stage (Jackson et al., 2010). Therefore EIF1AX and EIF2S3 are thus candidates for mediating, at least in part, the mechanism whereby erosion of XCI causes an increase in protein translational capacity. Interestingly, both the EIF1AX and EIF2S3 genes have previously been categorized as facultative XCI escapees (Belling et al., 2017; Zhang et al., 2013), meaning they are among a subset of X-linked genes that can escape transcriptional repression, despite the globally repressed state. This local increased gene dosage effect suggests that even female lines with normal XCI may differ in translational capacity from male cells with only a single X chromosome.

Our data show that erosion of XCI in human cells has the potential to cause major changes at the level of protein expression, which in turn could have important implications for disease progression and response to therapy in females. The potential clinical relevance is amplified by our finding that the expression of numerous autosomal genes also respond to erosion of XCI at the protein level. Many of these significantly increased proteins encoded on autosomes, such as ERK2, FYN, and CDK6, are linked to cancer and other diseases (Papa et al., 2019; Saito et al., 2010; Yang et al., 2017). It will also be of interest to analyze whether, or to what extent, the loss of XIST expression can display similar consequences *in vivo*, as it has been reported that multiple cell types and tissues have reduced or no XIST expression (Syrett et al., 2017; Wang et al., 2016).

## STAR★Methods

### Key resources table

**Table:** 

REAGENT or RESOURCE	SOURCE	IDENTIFIER
**Deposited data**		

Proteomic data	PRIDE repository	PRIDE:PXD010557
RNaseq data	ENA repository	ENA:PRJEB7388

**Experimental models: Cell lines**		

Human induced pluripotent stem cells	HipSci (https://www.hipsci.org/)	RRID:SCR_003909

**Software and algorithms**		

MaxQuant (v.1.6.3.3)	Tyanova et al., 2016	RRID:SCR_014485
Salmon (v.0.8.2)	Patro et al., 2017	RRID:SCR_017036
STAR (v.020201)	Dobin et al., 2013	RRID:SCR_015899
Trim Galore	Babraham Bioinformatics	RRID:SCR_011847
R (v.3.6.0)	R Project	RRID:SCR_001905
Limma (v.3.7)	Ritchie et al., 2015	RRID:SCR_010943
Qvalue (v.2.10.0)	Bioconductor	RRID:SCR_001073
edgeR	Robinson et al., 2010	RRID:SCR_012802
featureCounts (v.1.6.0)	Bioconductor	RRID:SCR_012919
Jalview (v.2.11.1.3)	Waterhouse et al., 2009	RRID:SCR_006459

### Resource availability

#### Lead contact

Further information and requests for resources should be directed to and will be fulfilled by the lead contact, Angus I. Lamond (a.i.lamond@dundee.ac.uk)

#### Materials availability

This study did not generate new unique reagents.

#### Data and code availability

The mass-spectrometry dataset, PXD010557, supporting the current study is available in PRIDE (https://www.ebi.ac.uk/pride/archive/projects/PXD010557). The RNaseq dataset, PRJEB7388, supporting the current study is available in the ENA project (https://www.ebi.ac.uk/ena/browser/view/PRJEB7388).

### Experimental model and subject details

All lines included in this study are part of the HipSci resource and were reprogrammed from primary fibroblasts as previously described (Kilpinen et al., 2017). Out of the total of more than 800 iPSC lines available within the HipSci resource (https://www.hipsci.org), 120 derived from healthy donors and with proteomic analysis were used in this study. All lines derived from healthy female donors (subset of 74 iPSC lines) were then used for the XCI analysis and included all lines derived from healthy male donors (subset of 46 iPSC lines) for the dosage compensation analysis.

### Method details

#### TMT Sample preparation

The data presented here is a subset of the total HipSci proteomics dataset (Brenes et al., 2019; Mirauta et al., 2020). For protein extraction, iPSC cell pellets were washed with ice cold PBS and redissolved immediately in 200 μL of lysis buffer (8 M urea in 100 mM triethyl ammonium bicarbonate (TEAB)) and mixed at room temperature for 15 minutes. The DNA content of the cells was sheared using ultrasonication (6 X 20 s on ice). The proteins were reduced using tris-carboxyethylphosphine TCEP (25 mM) for 30 minutes at room temperature, then alkylated in the dark for 30 minutes using iodoacetamide (50 mM). Total protein was quantified using the EZQ assay (Life Technologies). The lysates were diluted with 100 mM TEAB 4-fold for the first digestion with mass spectrometry grade lysyl endopeptidase, Lys-C (Wako, Japan), then further diluted 2.5-fold before a second digestion with trypsin. Lys-C and trypsin were used at an enzyme to substrate ratio of 1:50 (w/w). The digestions were carried out overnight at 37°C, then stopped by acidification with trifluoroacetic acid (TFA) to a final concentration of 1% (v:v). Peptides were desalted using C18 Sep-Pak cartridges (Waters) following manufacturer’s instructions.

For tandem mass tag (TMT)-based quantification, the dried peptides were re-dissolved in 100 mM TEAB (50 ml) and their concentration was measured using a fluorescent assay (CBQCA, Life Technologies). 100 mg of peptides from each cell line to be compared, in 100 mL of TEAB, were labeled with a different TMT tag (20 mg ml−1 in 40 mL acetonitrile) (Thermo Scientific), for 2 h at room temperature. After incubation, the labeling reaction was quenched using 8 mL of 5% hydroxylamine (Pierce) for 30 min and the different cell lines/tags were mixed and dried in vacuo.

The TMT samples were fractionated using offline high-pH reverse-phase (RP) chromatography: samples were loaded onto a 4.6 × 250 mm Xbridge BEH130 C18 column with 3.5-mm particles (Waters). Using a Dionex bioRS system, the samples were separated using a 25-min multistep gradient of solvents A (10 mM formate at pH 9) and B (10 mM ammonium formate pH 9 in 80% acetonitrile), at a flow rate of 1 mL min−1. Peptides were separated into 48 fractions, which were consolidated into 24 fractions. The fractions were subsequently dried and the peptides re-dissolved in 5% formic acid and analyzed by LC–MS/MS.

#### TMT LC–MS/MS

The data presented here is a subset of the total HipSci proteomics dataset (Brenes et al., 2019; Mirauta et al., 2020). Samples were analyzed using an Orbitrap Fusion Tribrid mass spectrometer (Thermo Scientific), equipped with a Dionex ultra-high-pressure liquid-chromatography system (RSLCnano). RPLC was performed using a Dionex RSLCnano HPLC (Thermo Scientific). Peptides were injected onto a 75 μm × 2 cm PepMap-C18 pre-column and resolved on a 75 μm × 50 cm RP- C18 EASY-Spray temperature-controlled integrated column-emitter (Thermo Scientific), using a four-hour multistep gradient from 5% B to 35% B with a constant flow of 200 nL min−1. The mobile phases were: 2% ACN incorporating 0.1% FA (solvent A) and 80% ACN incorporating 0.1% FA (solvent B). The spray was initiated by applying 2.5 kV to the EASY-Spray emitter and the data were acquired under the control of Xcalibur software in a data-dependent mode using top speed and 4 s duration per cycle. The survey scan was acquired in the orbitrap covering the m/z range from 400 to 1,400 Thomson with a mass resolution of 120,000 and an automatic gain control (AGC) target of 2.0 × 105 ions. The most intense ions were selected for fragmentation using CID in the ion trap with 30% CID collision energy and an isolation window of 1.6 Th. The AGC target was set to 1.0 × 104 with a maximum injection time of 70 ms and a dynamic exclusion of 80 s.

During the MS3 analysis for more accurate TMT quantifications, 5 fragment ions were co-isolated using synchronous precursor selection using a window of 2 Th and further fragmented using HCD collision energy of 55%. The fragments were then analyzed in the orbitrap with a resolution of 60,000. The AGC target was set to 1.0 × 105 and the maximum injection time was set to 105 ms.

#### Ribo Mega-SEC iPSC lines cell culture

For the Ribo Mega-SEC analyses 4 iPSC lines with High XIST RNA levels (‘iiyk_2′, ‘iiyk_4’, ‘nufh_3′ and nufh_4’) and 3 lines with Low XIST RNA levels (‘fawm_4’, ‘bawa_1’ and ‘aizi_3′) were used. The lines were maintained in TESR medium (Ludwig et al., 2006), supplemented with FGF2 (Peprotech, 30 ng/ml) and noggin (Peprotech, 10 ng/ml), on growth factor reduced geltrex basement membrane extract (Life Technologies, 10 μg/cm^2^) coated dishes at 37°C in a humidified atmosphere of 5% CO_2_ in air.

Cells were routinely passaged twice a week as single cells, using TrypLE select (Life Technologies) and replated in TESR medium that was further supplemented with the Rho kinase inhibitor Y27632 (Tocris, 10 μM), to enhance single cell survival. Twenty-four hours after replating, Y27632 was removed from the culture medium. For proteomic analyses, cells were plated in 100 mm geltrex coated dishes at a density of 5x10^4^ cells cm^-2^ and allowed to grow to for 3 days, until confluent, with daily medium changes.

#### Ribo Mega-SEC

Ribo Mega-SEC for the separation of polysomes and ribosomal subunits using size exclusion chromatography was performed as previously reported (Yoshikawa et al., 2018), with a slight modification. Briefly, 2.5 × 10^6^ cells were washed once with ice-cold PBS, scraped in ice-cold PBS and collected by centrifugation at 500 g for 5 min (all centrifugations at 4°C). The cells were lysed by vortexing for 10 s in 250 μL of polysome extraction buffer (20 mM HEPES-NaOH (pH 7.4), 130 mM NaCl, 10 mM MgCl_2_, 5% glycerol, 1% CHAPS, 0.2 mg/ml heparin, 2.5 mM DTT, 20 U SUPERase In RNase inhibitor, cOmplete EDTA-free Protease inhibitor), incubated for 15 min on ice, and centrifuged at 17,000 g for 10 min. Supernatants were filtered through 0.45 μm Ultrafree-MC HV centrifugal filter units (Millipore).

Using a Dionex Ultimate 3,000 Bio-RS uHPLC system (Thermo Fisher Scientific), a SEC column (Agilent Bio SEC-5, 2,000 Å pore size, 7.8 × 300 mm with 5 μm particles) was equilibrated with three column volumes of filtered SEC buffer (20 mM HEPES-NaOH (pH 7.4), 60 mM NaCl, 10 mM MgCl_2_, 0.3% CHAPS, 0.2 mg/ml heparin, 2.5 mM DTT, 5% glycerol) (all column conditioning and separation at 5°C) and 100 μL of 10 mg/ml of filtered bovine serum albumin (BSA) solution diluted by PBS was injected once to block the sites for non-specific interactions. After monitoring the column condition by injecting standards, including 10 μL of 10 mg/mL BSA solution and 5 μL of HyperLadder 1 kb (BIOLINE), 200 μL of the filtered cell lysates was injected onto the pre-equilibrated SEC column. The flow rate was 0.4 mL/min and the chromatogram was monitored by measuring UV absorbance at 215, 260 and 280 nm with a 1 Hz data collection rate by the Diode Array Detector.

#### RNA-seq data processing

Raw RNA-seq data were obtained from the ENA project: PRJEB7388. CRAM files were merged on a sample level and converted to a single FASTQ file per sample. Sequencing reads were trimmed to remove adapters and low-quality bases (Trim Galore!), followed by read alignment using STAR (v.020201) (Dobin et al., 2013), using the two-pass alignment mode and the default parameters as proposed by ENCODE (c.f. STAR manual). All alignments were relative to the GRCh37 reference genome, using ENSEMBL 75 as transcript annotation (Zerbino et al., 2018).

Samples with low quality RNA-seq were discarded if they had either less than 2 billion bases aligned, had less than 30% coding bases, or had a duplication rate higher than 75%. Gene-level RNA expression was quantified from the STAR alignments using featureCounts (Liao et al., 2014) (v1.6.0), which was applied to the primary alignments using the “-B” and “-C” options in stranded mode, using the ENSEMBL 75 GTF file. Quantifications per sample were merged into an expression table using the following normalization steps. First, gene counts were normalized by gene length. Second, the counts for each sample were normalized by sequencing depth using the edgeR (Robinson et al., 2010) adjustment. Transcript isoform expression was quantified directly from the (unaligned) trimmed reads using Salmon (Patro et al., 2017) (v0.8.2), using the ‘–seqBias’, ‘–gcBias’ and ‘VBOpt’ options in ‘ISR’ mode to match our inward stranded sequencing reads. The transcript database was built on transcripts derived from ENSEMBL 75. The TPM values as returned by Salmon were combined into an expression table.

#### Allele specific analysis

Allele-specific quantification of expression from the X chromosome was calculates using RNA-Seq reads mapping to the X chromosome. Allele-specific counts were obtained from SNPs present in DBSNP using GATK ReadCounter with the command ‘GenomeAnalysisTk.jar -T ASEReadCounter -U ALLOW_N_CIGAR_READS –minMappingQuality 10 –minBaseQuality 2′, restricted to SNPs which were known to be heterozygous in the analyzed sample. The allele-specific fraction of expression was defined as the fraction of transcript reads mapping to the less expressed allele, restricting to heterozygous X chromosome SNPs with at least 20 overlapping reads. These fractions were then averaged across SNPs at a whole-chromosome level (Figures 2A and 2C), and for individual genes (Figures 6A and 6D). Note that, for a given gene in a given sample, this quantification could only be performed when the donor for that sample carries a heterozygous common variant in that gene. This reduced the number of samples for which allele-specific expression could be computed for each gene.

#### Primary versus secondary allele

The primary allele for each iPSC line is defined as the allele with the highest number of transcript reads mapping to it. Conversely the secondary allele is defined as the allele with the lowest number of transcript reads mapped to it.

#### Proteomics data processing

The TMT-labeled samples were collected and analyzed using Maxquant (Cox and Mann, 2008; Tyanova et al., 2016) v. 1.6.3.3. The FDR threshold was set to 1% for each of the respective Peptide Spectrum Match (PSM) and Protein levels. The data were searched with the following parameters; type was set to Reporter ion MS3 with 10plex TMT, stable modification of carbamidomethyl (C), variable modifications, oxidation (M), acetylation (protein N terminus), deamidation (NQ), Glutamine to pyro-glutamate (N terminus), with a 2 missed tryptic cleavages threshold, reporter mass tolerance set to 0.03 ppm. Minimum peptide length was set to 7 amino acids. Proteins and peptides were identified using UniProt (SwissProt December 2018). Run parameters have been deposited to PRIDE (Perez-Riverol et al., 2019) along with the full MaxQuant quantification output (PDX010557).

#### Razor + unique peptides

Peptides that are unique to a single protein sequence are known as “unique peptides” and peptides that are shared between multiple protein sequences are known as “shared peptides.” Within MaxQuant, shared peptides are assigned to a single protein group, following Occam’s Razor. The number of unique peptides, plus the number of shared peptides used for the quantification of a protein group, is referred to as Razor + unique peptides.

#### iPSC Copy number generation

Protein copy numbers were calculated using the proteomic ruler (Wiśniewski et al., 2014) and using the MS3 reporter intensity. An additional batch correction factor for each TMT experiment was applied as previously described (Brenes et al., 2019).

#### Protein content

The protein content for all iPSC lines was calculated based on the copy numbers. The molecular weight of each protein (converted to picograms) was multiplied by the number of copies for the corresponding protein and this was then summed for all proteins across each line to calculate the protein content.

#### Chromosome mapping

To map gene products to their specific chromosomes, we utilized the UniProt (The UniProt Consortium, 2017) protein-chromosome mapping service. We used their output to produce a list of unique proteins for each specific chromosome. Subsequently, we mapped the proteins detected in our iPSC dataset to their corresponding chromosomes, based on the UniProt mapping file.

#### X-inactivation stratification and analysis

Based on the RNaseq data, 74 iPSC lines were classified into 3 distinct categories, based on XIST expression. 30 iPSC lines where XIST expression was < 1 Log_2_ TPM were classified as ‘Low XIST’. 35 iPSC lines where XIST expression was higher than 2.75 Log_2_ TPM were classified as High XIST and the remaining 9 lines were classified as ‘Medium’ XIST.

#### High XIST filtering

The High XIST population contained two proteomic experiments, PT7422 and PT6386, contributing a large number of High XIST replicates within their 10-plex TMT experiment. As the maximum number of replicates per 10-plex within the Low XIST group was 4, we performed hierarchical clustering to reduce the number of lines contributed by PT7422 and PT6386 to a maximum of 4, in order to minimize batch effects. The final number of lines with High XIST, post filtering, was 26.

#### GO Enrichment analysis

All of the GO enrichment analyses were done using Panther (Mi et al., 2017) and used the 8,511 proteins that were detected in both the RNaseq and proteomics datasets as background. We performed a biological process overrepresentation analysis for all proteins that were significantly increased (q-value < 0.05), where the corresponding transcript was not significantly increased in expression. Furthermore, an additional biological process overrepresentation of significantly increased (q-value < 0.05) ribosome biogenesis proteins was carried out.

#### Hypergeometric analysis

The hypergeometric analyses were all done in R using the phyper function from the stats package (v.3.6.0). For this analysis, a subset of proteins (1,825), which were significantly increased in expression without a corresponding mRNA increase, were selected. We first looked at the number of proteins within the previous subset with a peptide count either greater than, or equal to, the 75^th^ percentile (26 peptides) and used phypher to determine hypergeometric p values. We then compared this result to the one produced by randomly selecting 1,825 proteins and repeating the previous process. This was done 100,000 times. We also looked at the number of proteins with copy numbers either greater than, or equal to, the 75^th^ percentile (407,724 copies) and used phypher to determine hypergeometric p values. We then compared this result to the one produced by randomly selecting 1,825 proteins and repeating the previous process. This was done 100,000 times.

#### 60S/40S ratio

The ratios were calculated by summing the copy numbers from all proteins of the 60S ribosomal subunit, divided by the sum of all copy numbers from the 40S ribosomal subunit, for each individual iPSC line.

#### UniProt to Ensembl mapping

Mapping of UniProt accessions to Ensembl gene identifiers was done in R using the “UniProt.ws” package version 2.24.1

#### Kinase map

The kinase map was generated within the Encyclopedia of Proteome Dynamics (Brenes et al., 2018) using the KinoViewer (Brenes and Lamond, 2019).

#### Sequence coverage maps

The sequence coverage maps for EIF1AX, EIF2S3 and RPS6KA3 were generated using Jalview (Waterhouse et al., 2009) version 2.11.1.3

### Quantification and statistical analysis

The proteomics data used for the analysis were obtained from the ProteinGroups.txt output of Maxquant (Cox and Mann, 2008; Tyanova et al., 2016) v. 1.6.3.3. Contaminants, reverse hits and ‘only identified by site’ proteins were excluded from analysis. Overall, we quantified 9,631 protein groups in at least one of the samples. For additional stringency and to reduce batch variation, only proteins with 3 or more ‘Razor + unique peptides’ were considered.

Fold changes and P values were calculated in R utilizing the bioconductor package LIMMA (Ritchie et al., 2015) version 3.7. FDR calculations were performed in R with the “qvalue” package version 2.10.0. For both the RNaseq and proteomics differential expression analyses, gene products with a Q-value ≤ 0.05 were considered significant. For comparisons looking at individual gene products or aggregated gene families, Welch’s t test was used. In this case results with a p value ≤ 0.05 were considered significant.

## Supplemental information

Document S1. Figures S1–S6

Table S1. Protein and RNA abundance, related to Figures 1, 2, 3, 4, 5, and 6 Protein copy numbers and transcript TPMs for all gene products across all iPSC cell lines.

Table S2. iPSC line TMT and XIST mapping, related to Figures 2, 3, 4, 5, and 6 Mapping of the different iPSC lines to TMT reporter channels as well as their XIST stratified population.

Table S3. Significantly increased X-linked gene products, related to Figure 3 X-linked proteins and transcripts that are significantly increased in expression within the Low XIST population.

Table S4. Protein and RNA differential expression, related to Figures 2, 3, 4, and 5 Full differential expression tables for the proteomic and transcriptomic data, including a merged sheet with both datasets integrated.

Document S2. Article plus supplemental information
