# Supplementary material for: Erosion of human X chromosome inactivation causes major remodeling of the iPSC proteome
Source: Cell Rep. 2021 Apr 27;35(4):109032. doi: 10.1016/j.celrep.2021.109032 (PMC8097692; doi:10.1016/j.celrep.2021.109032)
Supplement: Document S1. Figures S1–S6 [file mmc1.pdf]

Cell Reports, Volume *35*

Supplemental information

Erosion of human X chromosome inactivation causes major remodeling of the iPSC proteome

Alejandro J. Brenes, Harunori Yoshikawa, Dalila Bensaddek, Bogdan Mirauta, Daniel Seaton, Jens L. Hukelmann, Hao Jiang, Oliver Stegle, and Angus I. Lamond

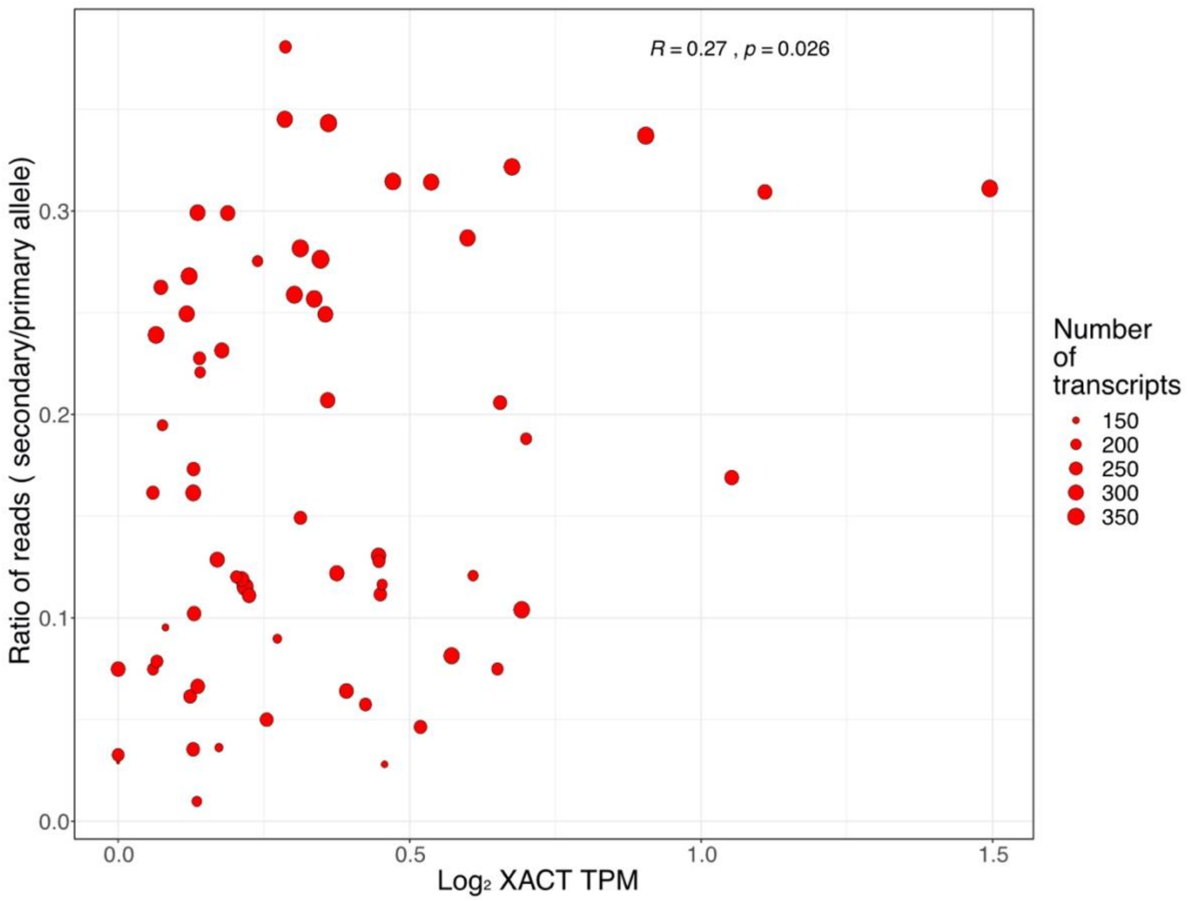

Figure S1 – XACT and XCI: (a) Scatter plot showing the fraction of reads derived from the secondary (lowest expressed) allele compared to the primary (highest expressed) allele for all X-linked transcripts vs the $\log_2$ XACT TPM for all healthy female lines. The size is determined by the number of transcripts used for the analysis. Related to Figure 2.

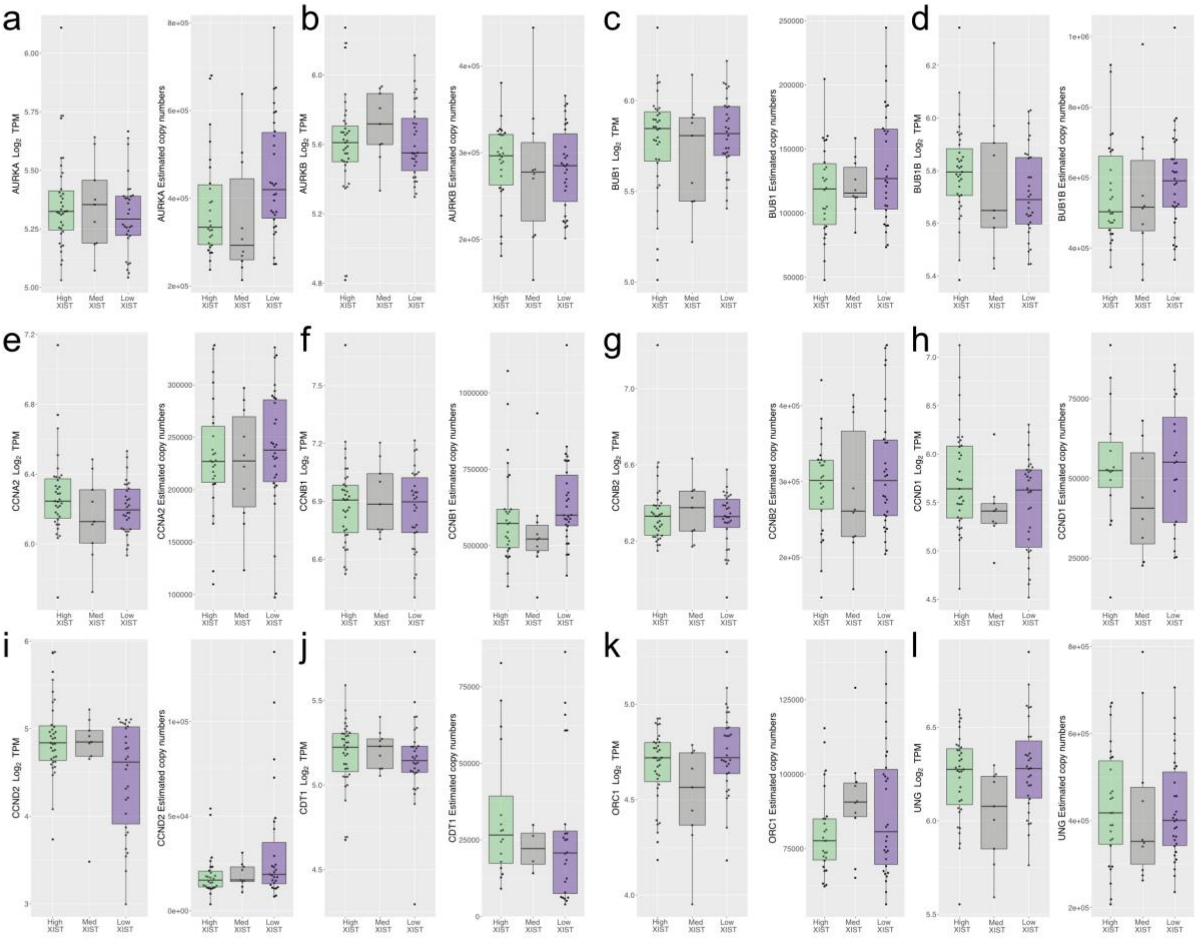

Figure S2 – Cell cycle markers: All box plots show the log_2 TPM and estimated protein copy numbers within the High, Medium and Low XIST populations for a gene product. The upper whisker extends from the hinge to the largest value no further than 1.5 * IQR from the hinge, the lower whisker extends from the hinge to the smallest value at most 1.5 * IQR of the hinge. (a) Boxplots for AURKA. (b) Boxplots for AURKB. (c) Boxplots for BUB1. (d) Boxplots for BUB1B. (e) Boxplots for CCNA2. (f) Boxplots for CCNB1. (g) Boxplots for CCNB2. (h) Boxplots for CCND1. (i) Boxplots for CCND2. (j) Boxplots for CDT1. (k) Boxplots for ORC1. (l) Boxplots for UNG. Related to Figure 3.

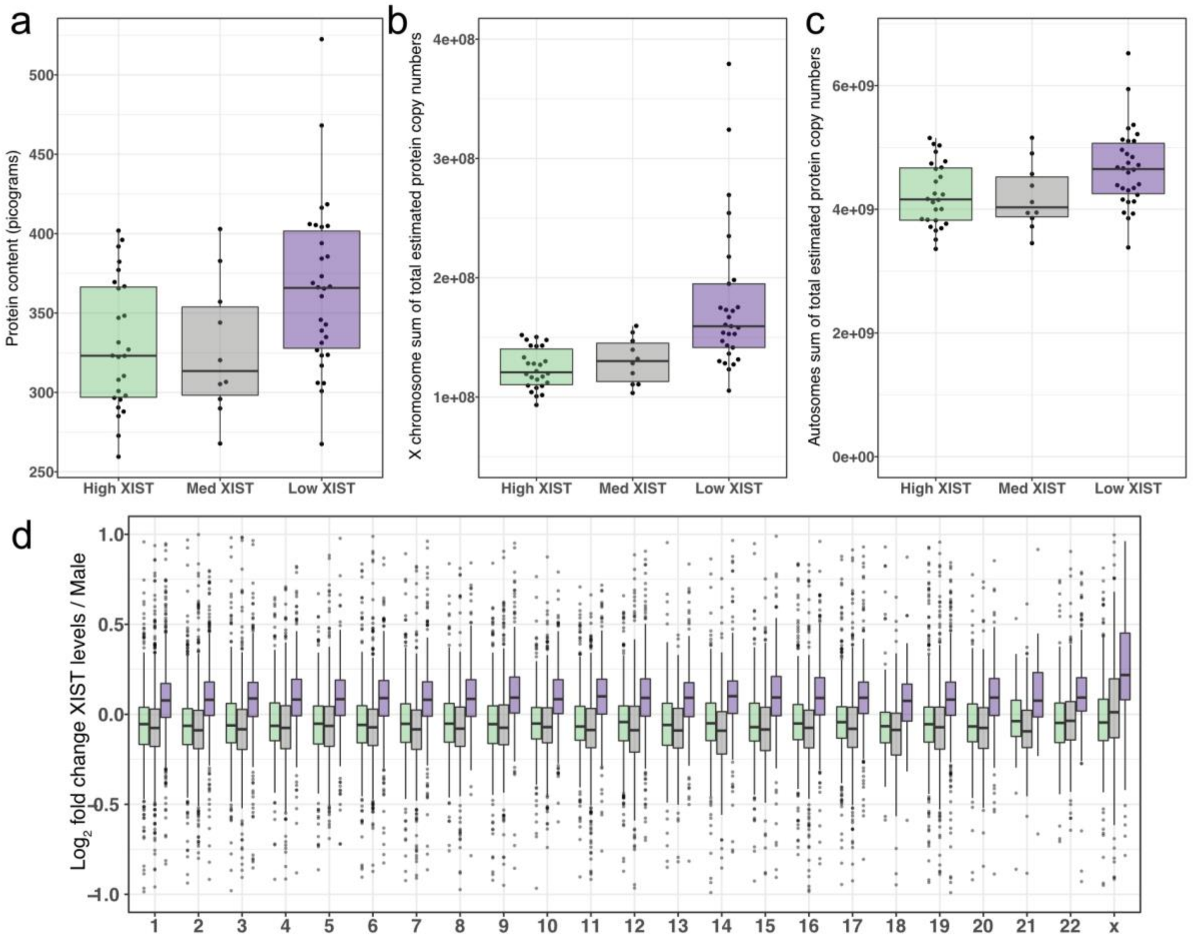

Figure S3 – High, Medium and Low XIST protein level data : For all box plots the upper whisker extends from the hinge to the largest value no further than 1.5 * IQR from the hinge, the lower whisker extends from the hinge to the smallest value at most 1.5 * IQR of the hinge. **(a)** Box plot showing the estimated protein content (see methods) for the High, Medium and Low XIST. **(b)** Boxplot showing the sum of protein copy numbers across the X chromosome for the High XIST, Medium and Low XIST lines. **(c)** Boxplot showing the sum of protein copy numbers across all autosomes for the High XIST, Medium and Low XIST lines. **(d)** Boxplot showing the median protein $\log_2$ fold change for the High, Medium and Low XIST populations when compared to the male lines across all chromosomes. Related to Figure 3.

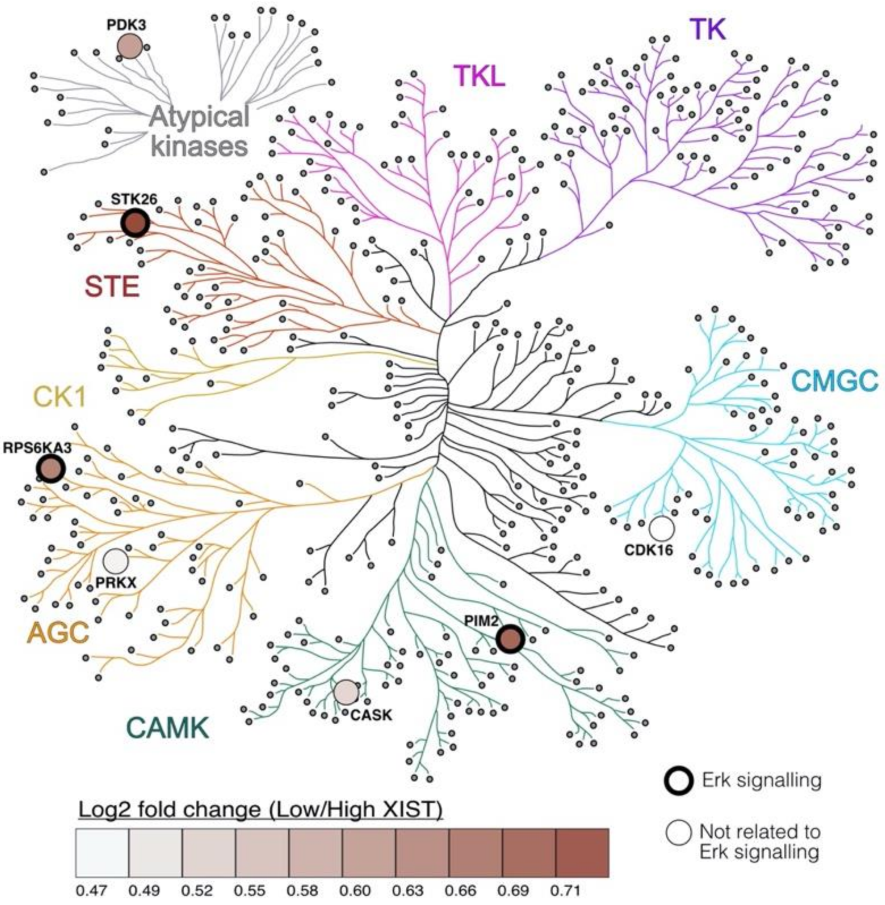

Figure S4 – X-linked Kinome: Protein level kinase map for all kinases showing the $\log_2$ fold change (Low/High XIST) for the kinases that were significantly in expression within the Low XIST population. Related to Figure 5.

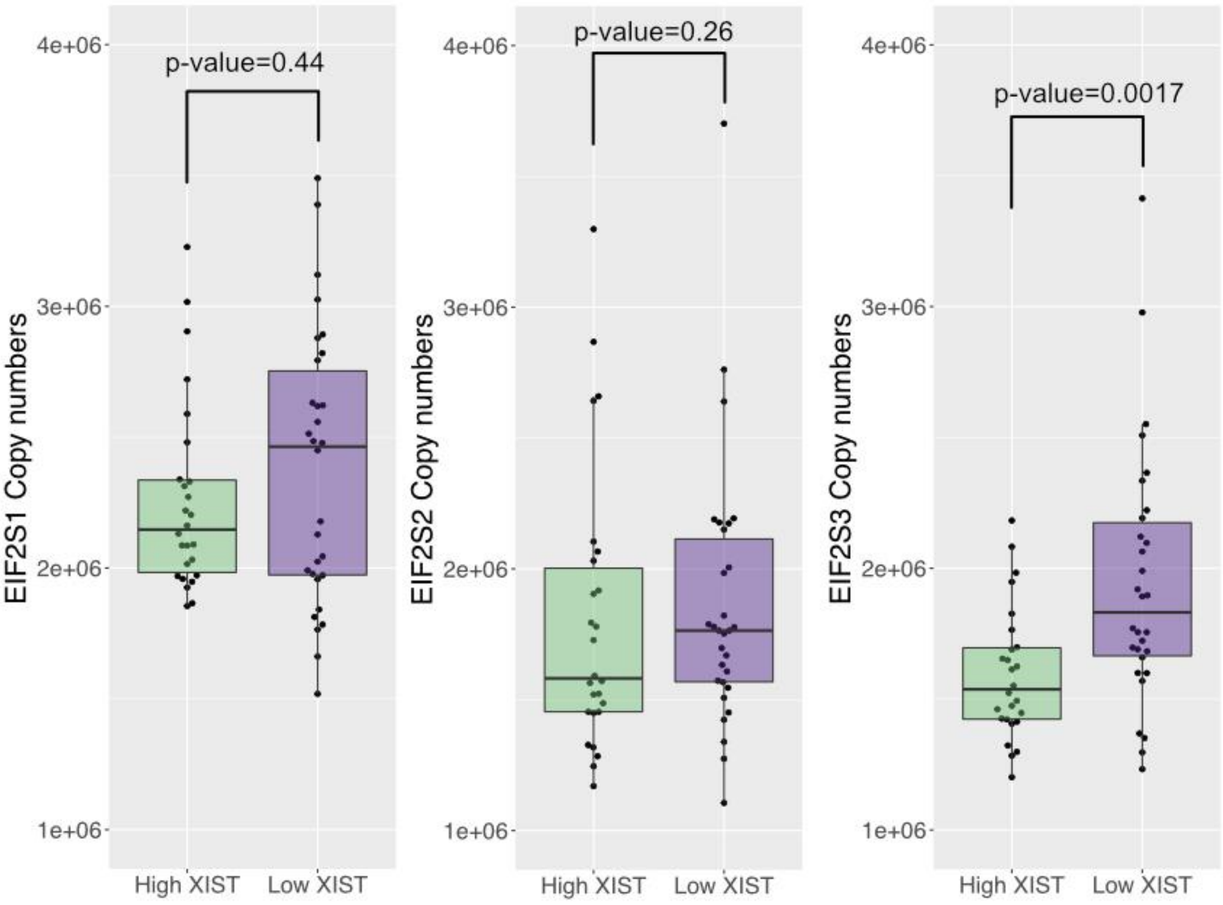

Figure S5 – eIF2 copy numbers: Box plot for the High and Low XIST populations showing the estimated copy numbers for EIF2S1, EIF2S2 and EIF2S3. The lower and upper hinges represent the 1st and 3rd quartiles. The upper whisker extends from the hinge to the largest value no further than 1.5 * IQR from the hinge, the lower whisker extends from the hinge to the smallest value at most 1.5 * IQR of the hinge. Related to Figure 6.

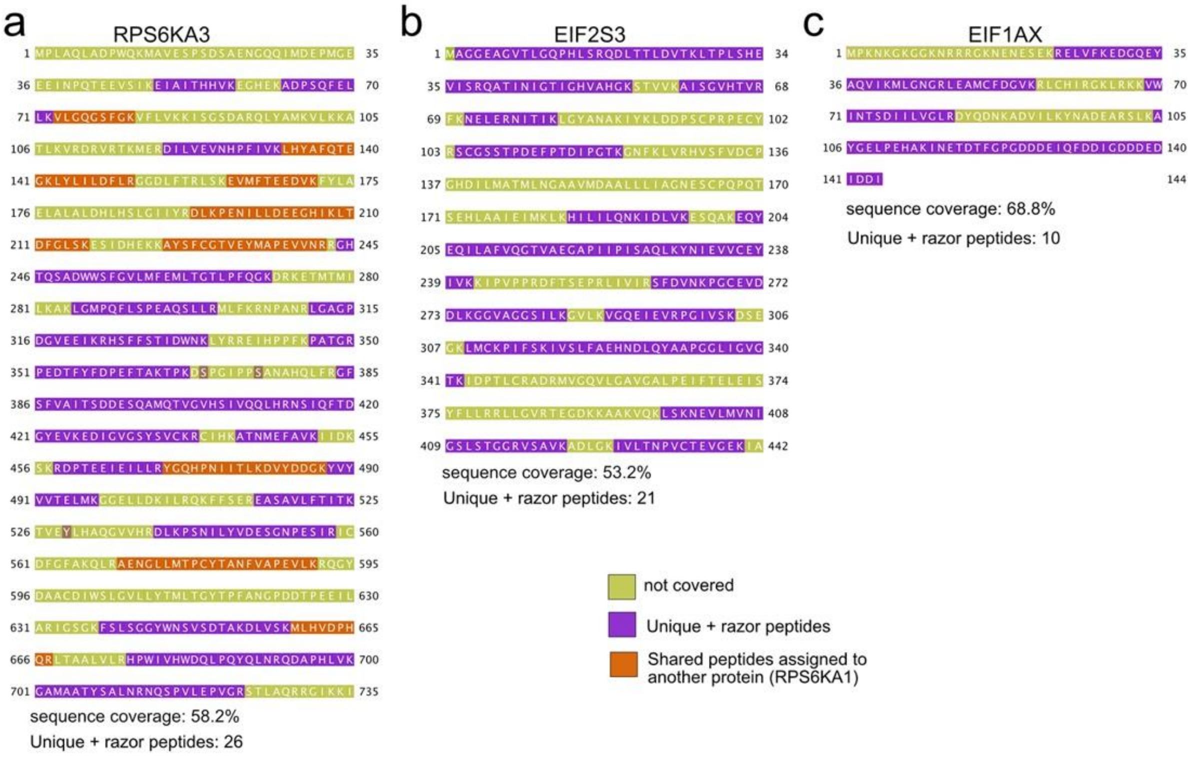

Figure S6 – Peptides and sequence coverage: (a) Map showing the amino acid sequence and MS derived peptide coverage for RPS6KA3. **(b)** Map showing the amino acid sequence and MS derived peptide coverage for EIF2S3. **(c)** Map showing the amino acid sequence and MS derived peptide coverage for EIF1AX. Related to Figures 5 & 6.
